# Evaluation of In Vitro Cytotoxic Potential of Avarol towards Human Cancer Cell Lines and In Vivo Antitumor Activity in Solid Tumor Models

**DOI:** 10.3390/molecules27249048

**Published:** 2022-12-19

**Authors:** Tatjana P. Stanojkovic, Marina Filimonova, Nadja Grozdanic, Slavica Petovic, Anna Shitova, Olga Soldatova, Alexander Filimonov, Jelena Vladic, Petr Shegay, Andrey Kaprin, Sergey Ivanov, Marina Nikitovic

**Affiliations:** 1 Department of Experimental Oncology, Institute of Oncology and Radiology of Serbia, 11000 Belgrade, Serbia; 2 A. Tsyb Medical Radiological Research Center, Federal State Budget Institution National Medical Research Radiological Center of the Ministry of Healthcare of the Russian Federation, 249031 Obninsk, Russia; 3 Institute of Marine Biology, University of Montenegro, 85330 Kotor, Montenegro; 4 Faculty of Technology, University of Novi Sad, 21000 Novi Sad, Serbia; 5 National Medical Research Radiological Center of the Ministry of Health of the Russian Federation, 249030 Obninsk, Russia; 6 Peoples’ Friendship University of Russia, Medical Institute (RUDN University), 117198 Moscow, Russia; 7 Faculty of Medicine, University of Belgrade, 11000 Belgrade, Serbia

**Keywords:** avarol, *Dysidea avara*, Ehrlich carcinoma, cervical cancer, cytotoxicity

## Abstract

The goal of this study was to determine the activity in vitro and in vivo of avarol, a sesquiterpene hydroquinone originating from the *Dysidea avara* sponge from the south Adriatic Sea, against different cancer cell lines and two types of mouse carcinoma. To investigate the in vitro cytotoxicity, a human cervix adenocarcinoma cell line (HeLa), human colon adenocarcinoma (LS174), human non-small-cell lung carcinoma (A549), and a normal human fetal lung fibroblast cell line (MRC-5) were used. The in vivo antitumor activity was investigated against two transplantable mouse tumors, the Ehrlich carcinoma (EC) and cervical cancer (CC-5). The effect of avarol on cancer cell survival, which was determined by the microculture tetrazolium test, confirmed a significant in vitro potency of avarol against the investigated cell lines, without selectivity towards MRC-5. The highest cytotoxicity was exhibited against HeLa cancer cells (10.22 ± 0.28 μg/mL). Moreover, potent antitumor activity against two tumor models was determined, as the intraperitoneal administration of avarol at a dose of 50 mg/kg resulted in a significant inhibition of tumor growth in mice. After three administrations of avarol, a 29% inhibition of the EC growth was achieved, while in the case of CC-5, a 36% inhibition of the tumor growth was achieved after the second administration of avarol. Therefore, the results indicate that this marine sesquiterpenoid hydroquinone could be a promising bioactive compound in the development of new anticancer medicine.

## 1. Introduction

Marine organisms are a valuable source of active substances that could be used in medicine. Numerous pharmacological and biological properties originating from marine organisms have been established, such as antibacterial, anticoagulant, antidiabetic, antifungal, anti-inflammatory, antitumor, antimalarial, and antiplatelet activities [1,2]. 

Marine invertebrates, encompassing sponges, tunicates, bryozoans, and mollusks, represent one of the most productive sources of bioactive components. Namely, as a defense mechanism against predators, these organisms secrete secondary metabolites. Apart from the defensive function, these metabolites exert significant biological functions and interfere with the pathogenesis of a wide range of diseases. Sponges, which belong to the phylum Porifera, represent rich sources of components, including sesquiterpene quinones and hydroquinones, which possess established cytotoxic and antiproliferative properties [3]. The compounds isolated from sponges, such as avarol, avarone, nakijiquinone, bolinaquinone, illimaquinone, dysiquinol D, and their derivatives, could be used for the development of the new antitumor agents [4,5,6,7,8].

**Figure 1:**
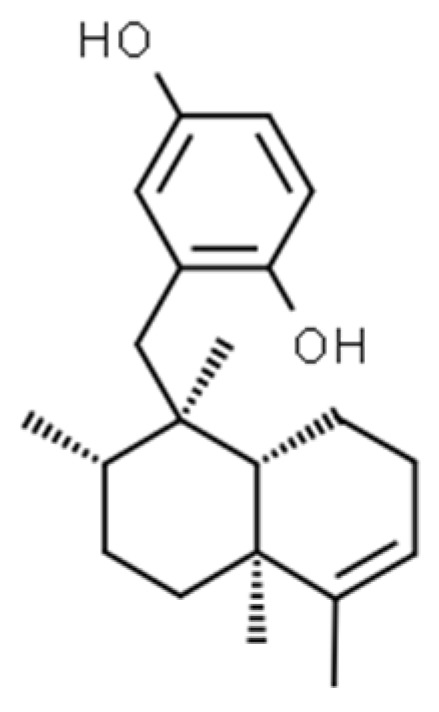
Chemical structure of avarol.

Avarol (Figure 1), a sesquiterpene hydroquinone, represents the main secondary metabolite isolated from the *Dysidea avara* sponge. It was determined that avarol and its derivatives demonstrate a broad spectrum of biological activities, including antiviral, antibacterial, antifungal, anti-inflammatory, acetylcholinesterase inhibitory, and antipsoriatic effects [3,9,10,11]. Moreover, due to its inhibitory effect on the synthesis of neutral lipids, avarol was suggested as an agent for the treatment of the metabolic syndrome [12]. In addition, the strong cytotoxic action of avarol was confirmed on several cell lines, such as pancreatic ductal adenocarcinoma cells, leukemia cell lines, and mouse lymphoma cells, as well as in vivo in a mouse model of L5178Y lymphoma [3,13,14]. Although the potential of avarol and its derivatives was established for the treatment of several types of cancer cells, further in vitro as well as in vivo research against different types of cancer are needed. Moreover, the aspects that need to be considered are the growing trend of cancer instances globally and the need for new agents that can potentially be used as a part of complex antitumor therapy. Moreover, natural compounds with anticancer properties represent an unlocked potential since they are renewable resources.

Lung cancer is among the most prevalent types of cancer in women and men. In terms of cancer diagnosis and cancer-related mortality, lung cancer is the most common malignancy, with 11.6% of all cancer diagnoses and 18.4% of cancer mortality [15]. Colorectal cancer represents the third most common cancer in men and the second most common cancer in women, with more than 1.9 million new cases in 2020 [16]. Furthermore, in 2020, over 604,000 women were diagnosed with cervical cancer [17]. Widely used chemotherapeutics and radiation treatments have many serious side effects [18]. Developing novel cancer treatments is essential for overcoming the undesirable effects of these treatments as well as tumor drug resistance [19]. Murine models are routinely used in studies of tumor pathogenesis and the research of novel anticancer agents. Ehrlich ascites or solid carcinoma (EC) is a spontaneous murine mammary adenocarcinoma that is a widely used model in cancer biology [20]. EC is originally a hyperdiploid undifferentiated carcinoma. The advantages of EC as a tumor model are that it is highly transplantable, as it does not have a tumor-specific transplantation antigen (TSTA), its fast rate of proliferation, and its 100% malignancy rate [21]. The solid form of EC emerged as an alternative tool for the reproduction of aggressive cancer models during preclinical research [22]. The transplantable mouse carcinoma CC-5 model is a murine cervical carcinoma induced by strong mutagen substance “X” (3-Chloro-4-(dichloromethyl)-5-hydroxy-2(5H)-furanone) that is used in experimental oncology [23].

Therefore, the aim of this study was to investigate the potential of avarol isolated from *Dysidea avara*, a sponge from the south Adriatic Sea, and to determine avarol’s (1) in vitro cytotoxic activity against various cancer cells, including a human cervix adenocarcinoma cell line (HeLa), human colon adenocarcinoma (LS174), human non-small-cell lung carcinoma (A549), and a normal human fetal lung fibroblast cell line (MRC-5), and (2) in vivo antitumor activity against two transplantable mouse tumors, EC and CC-5.

## 2. Results

### 2.1. Assessment of Cytotoxicity of Avarol In Vitro

Avarol was tested for in vitro cytotoxic activity against three human cancer cell lines (HeLa, LS174, and A549) and one normal human cell line (MRC-5). A standard chemotherapeutic, cisplatin, was used as a reference control compound. The cytotoxicity results are expressed as IC_50_ values in Table 1 and Figure S1 (Supplementary Data S1). Avarol exhibited the highest cytotoxic effect against HeLa cancer cells (10.22 ± 0.28 μg/mL), followed by the LS174 and A549 cancer cell lines. However, the cytotoxic effect was not selective to the normal MRC-5 cells (29.14 ± 0.41 μg/mL). Moreover, the coefficients of selectivity of avarol and cisplatin were calculated as the ratios between the IC_50_ values obtained on MRC-5 and malignant cells. Cisplatin’s selectivity coefficients were between 0.52 and 3.04, whereas for avarol they were between 0.83 and 2.85 (Table 2). It can be seen that the selectivity of avarol was the strongest towards Hela cells and was very similar to the selectivity of cisplatin.

### 2.2. Assessment of Antitumor Effect of Avarol In Vivo

Solid Ehrlich carcinoma is a convenient, reliable, and easily reproducible model for the initial assessment of antitumor potential. The development of solid EC in female F_1_ (CBA × C57BL/6j) mice in the current study was of the conventional nature inherent in this tumor model. By the 7th day after EC transplantation, tumor nodes were formed in all experimental animals (see Supplementary Material S2 and Table S1) and reached reliably measurable sizes. The volume of neoplasia in the animal groups at the beginning of the experiment was statistically indistinguishable (control group: 64.9 ± 19.4 mm^3^, test group: 73.1 ± 19.5 mm^3^, *p* = 0.49378).

In general, further administration (daily i.p. injections from the 7th to the 20th day after inoculation) of the solvent (a 1% hydroxypropylcellulose suspension) to the control animals did not significantly modify the development of EC. In the following two weeks, a 15–20-fold increase in the tumor nodes of the control animals was registered. Such dynamics of tumor node growth are typical for this model. At the terminal stage of the experiment, the tumor volume in the mice of the control group was 1183.2 ± 257.2 mm^3^. During these periods, the development of EC did not cause the deaths of tumor-bearing mice.

**Figure 2:**
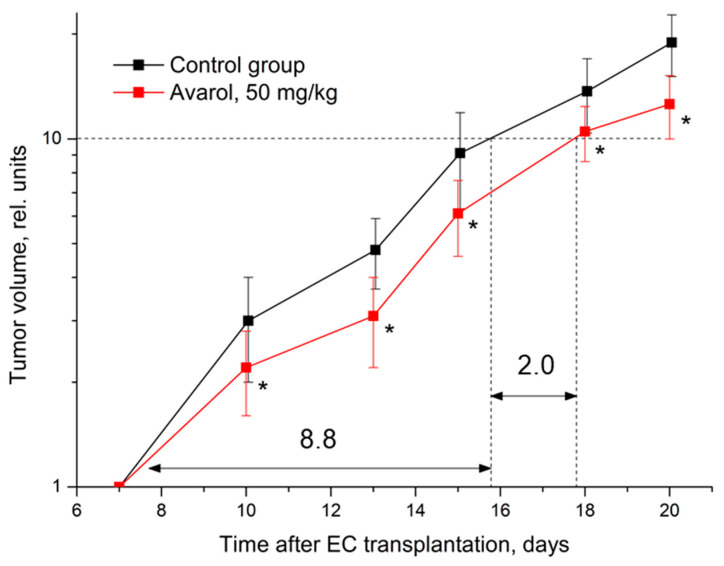
Influence of avarol on the dynamics of EC growth. Graphic deviations correspond to SD. *—statistically significant difference in tumor volumes compared to control. Dotted lines—assessment of the duration of growth retardation (days) in the time of a 10-fold increase in tumor volume. The arrows (and the numbers next to them) indicate a graphical estimate of the 10-fold increase in the volume of the tumor node.

The subchronic i.p. administration of avarol at 50 mg/kg caused a statistically significant antitumor effect on EC (Table 3, Figure 2). A significant 29% inhibition of neoplasia growth developed in mice after three injections of avarol. The continuation of avarol administration maintained a stable inhibition (25–35%) of EC growth in these mice. Overall, this caused a noticeable 23% increase in the time required to reach a 10-fold growth in tumor volume.

The method used for cervical cancer CC-5 transplantation to female CBA mice in this study caused a highly aggressive development of neoplasia. By the 7th day after CC-5 inoculation, a tumor node had formed in all experimental animals (Supplementary Material S2, Table S5) and had already reached a significant size. By the beginning of the experiment, the volumes of the neoplasia in the groups of animals were statistically indistinguishable (control group: 243.2 ± 80.9 mm^3^, experimental group: 275.1 ± 88.4 mm^3^, *p* = 0.74975). Over the next 11 days, an 8–11-fold increase in the tumor nodes of the control animals was registered. At the terminal stage of the experiment, the tumor volume in mice in the control group was 2022.5 ± 314.7 mm^3^. Such an aggressive development of this neoplasia during these periods resulted in the deaths of 17% of tumor-bearing animals in both groups.

**Figure 3:**
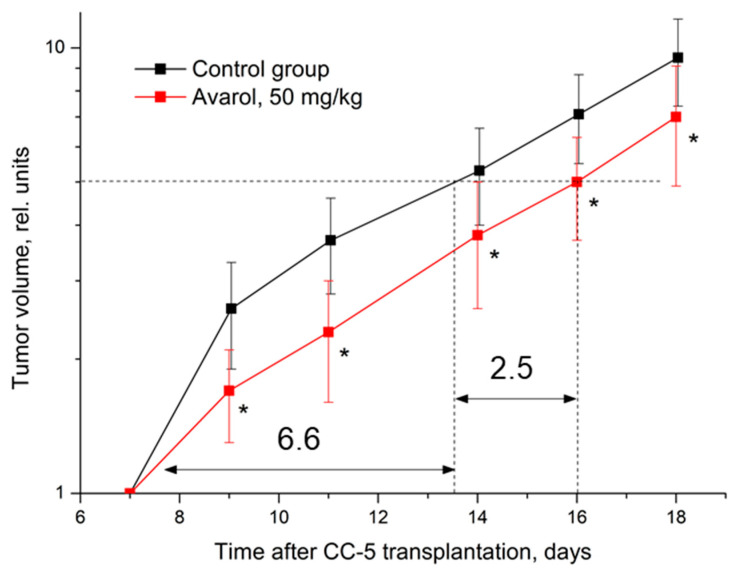
Influence of avarol on the dynamics of CC-5 growth. Graphic deviations correspond to SDs; *—statistically significant difference in tumor volume compared to control. Dotted lines—assessment of the duration of growth retardation (days) in the time of a five-fold increase in tumor volume. Arrows (and numbers next to them) indicate a graphical estimate of the time of a five-fold increase in the volume of the tumor node.

Despite the aggressiveness of this experimental neoplasia development, avarol also had a notable statistically significant antitumor effect on CC-5 (Table 4, Figure 3). In this case, a significant inhibition (36%) of tumor growth developed in mice after two avarol injections. Subsequent avarol administration maintained a stable CC-5 growth inhibition (within 28–37%) in these mice. Overall, this caused a significant increase of 38% in the time required to reach a five-fold growth in tumor volume.

**Figure 4:**
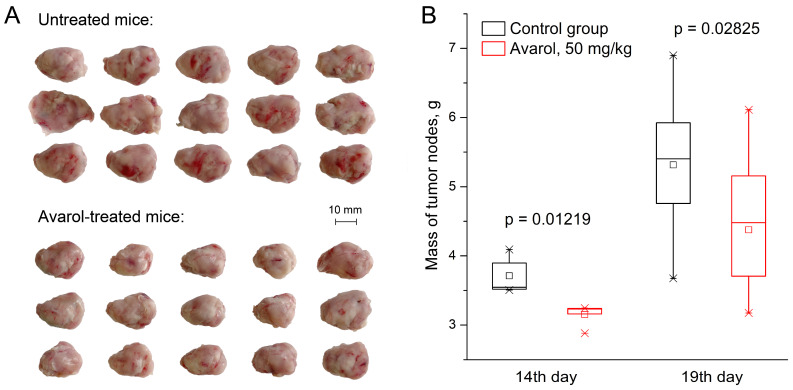
Influence of avarol on the masses of CC-5 tumor nodes on the 14th day (n = 5) and on the 19th day (n = 15) of observations. Panel (**A**)—the appearance of tumor nodes in the experimental groups on the 19th day of growth. Panel (**B**)—the distribution of the masses of tumor nodes in the experimental groups on the 14th and 19th days of growth. *p*—the levels of intergroup differences according to the Mann–Whitney U-test.

In addition, the antitumor effect of avarol against CC-5 in this experiment was also confirmed by statistically significant intergroup differences in the masses of tumor nodes in different periods of its course of use (Figure 4).

## 3. Discussion

The results of the in vitro cytotoxic action point to the good activity of this natural product against all tested cancer cell lines (HeLa, LS174, and A549). Although the demonstrated activity in vitro was promising, it was still below the activity of the chemotherapeutic drug cisplatin, but it was in accordance with the existing literature data [24]. Earlier studies had also shown the cytotoxic potential of avarol. Namba and Kodama demonstrated the cytotoxic effect on the following human cancer cell lines: pancreatic ductal adenocarcinoma (PDAC; Panc-1, PK1, and KLM1), lung cancer (A549), breast cancer (MCF7), osteosarcoma (U2OS), colorectal cancer (HCT116), and gastric cancer (AGS). The study also suggested the possible mechanism of avarol’s cytotoxic action, as the avarol-induced apoptosis in PDAC cells was activated by endoplasmic reticulum stress responses via the PERK-eIF2α-CHOP signaling pathway [14]. The induction of apoptosis and the inhibitory activity against the panel of tyrosine kinases were also established for the ethanolic extract of *D. avara* [25]. In addition, avarol exhibited antimitotic activity towards L5178Y cells, preventing the polymerization of proteins of brain microtubules. Moreover, stopping the process of the mitosis of cells is critical in chemotherapeutic procedures [13]. In addition, it was found that avarol can induce in vitro DNA strand breakage in Friend erythroleukemia cells in a dose-based manner [26]. It was suggested that the DNA damage and the consequential manifestation of cytotoxicity were most likely caused by the generation of oxygen radicals [3].

Furthermore, the selectivity of avarol towards the normal MRC-5 cell line was not recorded. However, the literature data showed the pronounced selectivity of avarol towards some other noncancer cell lines, including human fibroblast (IMR90 and HFL1), human embryonic kidney (HEK293), mouse embryonic fibroblast (MEF), human fibroblasts, and human gingival cells [13,14].

Avarol and its analogues in vivo are known to have antitumor cytostatic activity against leukemia cells in mice when administered intraperitoneally [13,27]. A previous in vivo study by Müller et al. showed that avarol’s chemotherapeutic activity increases the median life span of L5178Y-lymphoma-bearing mice [13]. Further experiments in our study demonstrated that avarol, besides its cytotoxic in vitro effects, had in vivo antitumor effects on two different types of mouse carcinoma (EC and CC-5). Thus, two solid transplantable tumors of different histogeneses were used in mice. It was shown that avarol, with a subchronic parenteral administration at a safe dose of 50 mg/kg, which is 1/5 of the LD_50_ [13], had a statistically significant antitumor effect. This effect was manifested in the pronounced and stable inhibition (25–35%) of neoplasia growth. Avarol, in the EC-inoculated group of mice, exerted a significant antitumor effect, extending the time required to reach a 10-fold growth in the tumor volume. In the control group of mice inoculated with highly aggressive cervical cancer for 11 days, the tumors increased in size by 8–11-fold, while in the group treated with avarol injections, a significant inhibition of tumor growth was recorded. Overall, the CC-5 mouse group treated with avarol had a significant increase in the time required for five-fold tumor volume growth. Therefore, the administration of avarol resulted in a significant reduction in the solid tumors’ mass development, indicating that the properties of avarol are effective in terminating tumor cells. For these reasons, this marine bioactive product can be used in the development of medicine for cancer therapy and curative chemotherapy.

In its chemical structure, avarol possesses a sesquiterpene skeleton and a reactive hydroquinone moiety. To determine which part of the molecule was responsible for the bioactivity, Kwatra tested the cytotoxic effect of polygodial, which has a chemical similarity with the avarol terpenoid moiety, towards U-251 MG cells. Considering that polygodial did not show activity against the investigated cells, it was concluded that the hydroquinone part of avarol was responsible for the cytotoxic activity [28]. In addition, Orhan et al. [29] suggested that the sesquiterpene hydroquinone accounted for the enzyme inhibitory activity of the *D. avara* extract due to the interference in the reactive oxygen species production and the redox status of cells. Avarol could impact the production of reactive oxygen species and, in that way, cause the induced cell death of lymphoma cells [26].

Conventional anticancer drugs usually target only the main pathway in the tumor, which can allow cells to survive and lead to new tumor growth. A potentially effective approach to treatment and the prevention of the development of adaptive resistance could be targeting the main and supporting pathways simultaneously [30]. Furthermore, conventional therapies, such as cisplatin, have serious side effects [31]. On the other hand, a two-week course of therapy with avarol did not cause negative manifestations or death in tumor-bearing animals. Therefore, natural compounds such as avarol that exhibit fairly pronounced antitumor effects and can be characterized as safer and cheaper with low toxicity and good tolerability [32] can represent a significant addition to the combined chemotherapy of tumors [30]. However, it is necessary to evaluate the acute and chronic toxicity of avarol in future studies to confirm the safety of its application as well as its low toxicity and good tolerance. Therefore, future research should be directed towards comparative studies of the efficacy, tolerability, and mechanism of action of avarol and conventional drugs such as cisplatin, considering the potentially different mechanisms of action. Moreover, achieving a better selectivity of avarol represents one of the future goals, which could potentially be gained through improvements in the biotechnological procedures for the isolation of avarol from sponges.

## 4. Materials and Methods

### 4.1. Avarol

Avarol was isolated from the extract of the sponge *Dysidea avara*, collected in the Bay of Kotor in the Adriatic Sea (Montenegro), according to Müller et al. [33]. Moreover, physical and chemical characterizations of avarol were previously described [33,34].

### 4.2. Drugs and Solutions

The (3-(4,5-di-methyl-thiazol-2-yl)-2,5-diphenyl tetrazolium bromide (MTT) was dissolved (5 mg/mL) in phosphate-buffered saline (pH 7.2) and filtered (0.22 µm) before use. The RPMI 1640 cell culture medium, fetal calf serum, cisplatin, and MTT were purchased from Sigma-Aldrich, St. Louis, MO, USA.

### 4.3. Cytotoxic Activity

#### 4.3.1. Cell Lines

A human cervix adenocarcinoma cell line (HeLa), human colon adenocarcinoma (LS174), human non-small-cell lung carcinoma (A549), and a normal human fetal lung fibroblast cell line (MRC-5) (American Type Culture Collection, Manassas, VA, USA, SAD) were grown in RPMI-1640 medium (Sigma-Aldrich, USA). The medium was supplemented with 10% fetal bovine serum, L-glutamine, and penicillin-streptomycin (Sigma-Aldrich, USA). Cells were grown at 37 °C in a humidified atmosphere with 5% CO_2_ in the air.

#### 4.3.2. Treatment of Cell Lines

Stock solutions (100 mg/mL) of compounds diluted in dimethyl sulfoxide (DMSO) were dissolved in the corresponding media to the required working concentrations.

Target HeLa (2000 cells per well), LS174 (7000 cells per well), A549 (5000 cells per well), and MRC-5 (5000 cells per well) cells were seeded into wells of 96-well flat-bottomed microtiter plates. After cell adherence for 24 h, five double-diluted concentrations of the investigated compounds were added to the wells. The final concentrations applied to target cells were 200, 100, 50, 25, and 12.5 μg/mL, except in the control wells, where only the nutrient medium was added to the cells. The cell cultures were incubated for 72 h. The final concentration of the DMSO solvent never exceeded 0.5% and represented a nontoxic concentration for the cells. The experiment was performed in triplicate and repeated three times.

#### 4.3.3. Determination of Cell Survival - MTT Test

The effects of the compounds on cancer cell survival were determined by the MTT test according to Mosmann [35] with modification by Ohno and Abe [36]. Briefly, 20 µL of MTT solution (5 mg/mL PBS) was added to each well. Samples were incubated for 4 h at 37 °C in 5% CO_2_ and a humidified air atmosphere. Then, 100 µL of 10% sodium dodecyl sulfate (SDS) was added to extract the insoluble product formazan, resulting from the conversion of the MTT dye by viable cells. The number of viable cells in each well was proportional to the intensity of the absorbance (A) of light, which was then read in an ELISA plate reader (ThermoLabsystems Multiskan EX. Oxford, UK) at 570 nm. To determine cell survival (%), the A of a sample with cells grown in the presence of various concentrations of the investigated compounds was divided by the control optical density (the A of the control cells grown only in nutrient medium) and multiplied by 100. In each experiment, the A of the blank was always subtracted from the A of the corresponding sample with target cells. IC_50_ was defined as the concentration of an agent inhibiting cell survival by 50% compared with the vehicle-treated control. All experiments were conducted in triplicate. Data were collected from three independent experiments and are presented as means ± SDs.

### 4.4. Antitumor Activity

#### 4.4.1. Compounds Used in In Vivo Experiments

In the study, avarol was used in the form of a 0.5% suspension prepared with water for injection (OOO Dalhimpharm, Habarovsk, Russia) in the presence of 1% hydroxypropyl cellulose (hyprolose, Nippon Soda CO Ltd., Tokyo, Japan). In all experiments, avarol was injected daily intraperitoneally (i.p.) at a dose of 50 mg/kg—10 µL of suspension per 1 g of animal weight. The animals in the control groups received daily i.p. injections of an equivalent volume of a 1% suspension of hydroxypropyl cellulose. All injectable suspensions were prepared *ex tempore*.

#### 4.4.2. Animals

Female mice (F_1_ (CBA × C57BL6j), 2–2.5 months, 19–23 g and CBA, 2 months, 19–22 g) were used in this study. The animals were obtained from the nursery of the Scientific Centre for Biomedical Technologies of the Federal Medical and Biological Agency of Russia. Mice were kept in the vivarium of the A.F. Tsyb MRRC in T-3 cages under conditions of natural light with forced 16x/hour ventilation at a temperature of 18–20 °C and a relative humidity of 40–70%. The animals had free access to water and PK-120-1 rodent food (OOO Laboratorosnab, Russia). The work with laboratory animals was approved by the MRRC Ethical Commission (protocol number: 1-H-00025) and was performed in accordance with generally accepted norms of animal handling based on standard operating procedures adopted at the A. Tsyb MRRC and corresponding to the rules and requirements of European Convention ETS/STE No. 123 and the international GLP standard (OECD Guide 1:1998).

#### 4.4.3. Tumor Models

Two transplantable mouse tumors were used: Ehrlich carcinoma (EC) and cervical cancer (CC-5). The tumor strains were obtained from the tumor bank of the N.N. Blokhin National Medical Research Centre for Oncology of the Ministry of Health of Russia. Before tumor grafting, the hair on the lateral surface of the right thigh was removed using a ChroMini Type 1591 trimmer (Moser, Germany). The EC strain was transplanted into female F_1_ (CBA × C57BL/6j) mice by the subcutaneous injection of 2.5 × 10^6^ tumor cells in 0.3 mL of medium 199 (PanEco LLC, Moscow, Russia) to the lateral surface of the right thigh. The CC-5 strain was transplanted into female CBA mice by the injection of 100 mg of tumor tissue homogenate in 0.5 mL of medium 199 into the lateral surface of the right thigh.

#### 4.4.4. Experimental Schemes and the Evaluation of Effects

Two independent experiments were performed on the selected tumor models. In the first experiment, the effect of avarol on the development of solid EC was studied. For this purpose, 33 female F_1_ (CBA × C57BL/6j) mice were divided into two groups, the control (17 animals) and experimental (16 animals) groups, immediately after EC grafting. Then, from the 7th to the 20th day (14 days) following the tumor transplantation, the animals in the experimental group were intraperitoneally (i.p.) injected daily with an avarol suspension at a dose of 50 mg/kg, and the animals in the control group were injected with an equivalent volume of solvent. The effect of avarol on tumor development was assessed by the growth dynamics of the tumor. Starting 7 days after transplanting EC and then every 2–3 days, the linear dimensions of the tumor nodes in all animals were measured with a caliper, and their volumes were estimated with the approximation of $V=\left(a\cdot {\left(\left(b+c\right)/2\right)}^{2}\right)\times \left(\pi /6\right)$, where a, b, and c are the orthogonal diameters. We calculated the relative volumes of the tumors normalized to the tumor volume in the animal at the treatment start day, and the tumor growth inhibition index in the animals treated with avarol was calculated as: $TI_{i,t}=\left({\bar{V}}_{C,t}-V_{i,t}\right)/{\bar{V}}_{C,t}\times 100\%$, where $TI_{i,t}$ is inhibition index in the *i*-th animal at observation time *t*, ${\bar{V}}_{C,t}$ is the average relative tumor volume in the control group at observation time *t*, and $V_{i,t}$ is the relative tumor volume of the *i*-th animal at observation time *t*.

The impact of cisplatin was previously investigated and established in earlier studies [31,37]. Hence, a group that received only cisplatin was not included in the research. Additionally, therapy with avarol during a two-week period did not result in negative manifestations or death in tumor-bearing animals. Therefore, an additional control group of healthy animals was not included.

The manifestation and dynamics of the avarol effect on EC were assessed and compared by intergroup statistical comparisons of the relative volumes of tumors and indicators of tumor growth inhibition in different follow-up periods. The integral antitumor effect was evaluated by the duration of growth retention, which was estimated by the time of a 10-fold increase in the EC volume on the tumor growth curves. At the end of the experiment (the day after the last injection), the animals were removed from the experiment by cervical dislocation under ether anesthesia.

In the second experiment, the effect of avarol on the development of CC-5 was studied. For this purpose, 48 female CBA mice were divided into two groups immediately after CC-5 grafting: the control and experimental groups (24 animals in each group). Five mice from each group were selected for the morphological examination of tumor nodes on the 14th day. Then, from day 7 to 18 after the tumor transplantation, the animals in the experimental group received a daily avarol suspension at the dose of 50 mg/kg, i.p., and the animals in the control group received an equivalent volume of the solvent. The effect of avarol on the development of CC-5 was evaluated using the same methods as in the EC experiment. The integral antitumor effect was evaluated by the duration of growth retardation, which was estimated by the time for a five-fold increase in the CC-5 volume on the tumor growth curves as well as by the difference in the masses of tumor nodes in the experimental groups on the 14th and 19th days of the observation. Supplementary Data S2 provides full protocols for studying the effect of avarol on the growth of SEC/CC-5 in mice, including individual indicators of the SEC/CC-5 volume at different periods after transplantation in the mice of the experimental groups, individual indicators of the SEC/CC-5 relative growth in different periods after transplantation in the mice of the experimental groups, SEC/CC-5 growth inhibition rates in avarol-treated mice in different follow-up periods, and the effect of avarol on SEC/CC-5 growth in mice (Supplementary Data S2, Tables S1–S8).

Supplementary Data S3 provides full protocols for studying the effects of avarol on body weight in mice with SEC/CC-5 with individual indicators of the body weights of the mice of the experimental groups in different periods after SEC/CC-5 transplantation, individual indicators of the relative body weights of the mice of the experimental groups in different periods after SEC/CC-5 transplantation, and the effect of avarol on the dynamics of body weight in mice after SEC/CC-5 inoculation (Supplementary Data S3, Tables S9–S14).

### 4.5. Statistical Processing of the Data

Standard parameters of variance statistics were calculated for all experimental results, and their values in the text, tables, and figures are given as means ± standard deviations (M ± SD). The significance levels of intergroup differences in the indicators were assessed by the Mann–Whitney U-test. In all cases, the effects were considered statistically significant at the 5% level. Calculations were performed using the Statistica 10.0 software package (StatSoft Inc., Tulsa, OK, USA).

## 5. Conclusions

The results demonstrated the pronounced cytotoxicity of avarol against the investigated cancer cells. The highest cytotoxic activity was recorded against the HeLa cells, followed by the LS174 and A549 cancer cell lines, with the absence of selectivity towards normal MRC-5 cells. Moreover, when parenterally administered, avarol significantly reduced the growth of two solid transplantable tumors. Avarol administration maintained stable inhibitions of 25–35% and 28–37% of EC and CC-5 growth, respectively. Therefore, the in vitro and in vivo results strongly confirm avarol’s potential as a promising natural agent for the treatment of different cancers. It is essential to conduct further research that would clarify the molecular mechanism of the manifestation of the antitumor activity of avarol and other sponge-derived compounds and further investigate the different possibilities for achieving better selectivity.

## Figures and Tables

**Table 1:** Concentrations that induced 50% decreases in HeLa, LS174, A549, and MRC-5 cell survival rates. The samples were incubated with cells for 72 h.

Samples	HeLa	LS174	A549	MRC-5
IC_50_ (μg/mL)				
Avarol	10.22 ± 0.28 *	34.06 ± 3.03 *	35.27 ± 0.64 *	29.14 ± 0.41 *
Cisplatin	3.46 ± 0.25	20.38 ± 0.44	17.93 ± 0.88	10.52 ± 0.22

IC_50_ values were obtained from the results of the MTT assays of three independent experiments and are expressed as the means ± SDs (standard deviations). *—statistically significant difference compared to control, *p* < 0.05.

**Table 2:** Selectivity coefficient.

Selectivity Coefficient	HeLa	LS174	A549
Avarol	2.85	0.86	0.83
Cisplatin	3.04	0.52	0.59

Selectivity indices towards malignant cell lines, calculated as the ratio of the IC_50_ value for MRC-5 and the IC_50_ value for the corresponding cell line.

**Table 3:** Effect of avarol (50 mg/kg i.p. daily) on EC growth in female F1 (CBA×C57BL/6j) mice.

Time after Inoculation (Days)	Tumor Volume, Rel. Units (M ± SD) * Number of Observations (n)		Inhibition of Tumor Growth (M ± SD; %)
	Control	Avarol	
7	1.0 n = 17	1.0 n = 16	0.0
10	3.0 ± 1.0 n = 17	2.2 ± 0.6 ^a^ n = 16	28.8 ± 15.6
13	4.8 ± 1.1 n = 17	3.1 ± 0.9 ^b^ n = 16	35.6 ± 18.0
15	9.1 ± 2.8 n = 17	6.1 ± 1.5 ^b^ n = 16	32.9 ± 16.4
18	13.7 ± 3.3 n = 17	10.5 ± 1.9 ^b^ n = 16	24.1 ± 12.7
20	18.9 ± 3.8 n = 17	12.6 ± 2.6 ^c^ n = 16	33.2 ± 13.7

*—Indices for each animal were normalized to the initial (on the 7th day) tumor volume before the start of the experimental treatment. Different letters indicate significant intergroup differences according to the Mann–Whitney U-test: ^a^—*p* < 0.01; ^b^—*p* < 0.001. ^c^—*p* < 0.0001.

**Table 4:** The influence of avarol (50 mg/kg i.p. daily) on the dynamics of CC-5 growth in female CBA mice.

**Time after Inoculation (Days)**	Tumor Volume, Rel. Units (M ± SD) * Number of Observations (n)		**Inhibition of Tumor Growth (M ± SD; %)**
	**Control**	**Avarol**	
7	1.0 n = 24	1.0 n = 24	0.0
9	2.6 ± 0.7 n = 24	1.7 ± 0.4 ^a^ n = 24	36.4 ± 15.3
11	3.7 ± 0.9 n = 24	2.3 ± 0.7 ^a^ n = 24	37.4 ± 17.3
14	5.3 ± 1.3 n = 22	3.8 ± 1.2 ^b^ n = 22	29.8 ± 21.3
16	7.1 ± 1.6 n = 17	5.0 ± 1.3 ^b^ n = 16	30.7 ± 17.4
18	9.5 ± 2.1 n = 15	7.0 ± 2.1 ^c^ n = 15	27.8 ± 18.0

*—Indices for each animal were normalized to the initial (on the 7th day) tumor volume before the start of the experimental treatment. Different letters indicate significant intergroup differences according to the Mann–Whitney U-test: ^a^—*p* < 0.00001; ^b^—*p* < 0.001. ^c^—*p* < 0.01.

## Data Availability

Not applicable.

## Supplementary Materials

The following supporting information can be downloaded at: https://www.mdpi.com/article/10.3390/molecules27249048/s1.
