# Supplementary material for: Evaluation of In Vitro Cytotoxic Potential of Avarol towards Human Cancer Cell Lines and In Vivo Antitumor Activity in Solid Tumor Models
Source: Molecules. 2022 Dec 19;27(24):9048. doi: 10.3390/molecules27249048 (PMC9788264; doi:10.3390/molecules27249048)
Supplement: Supplementary file 1 [file molecules-27-09048-s001.zip › molecules-2015316-supplementary.pdf]

Supplementary material S1

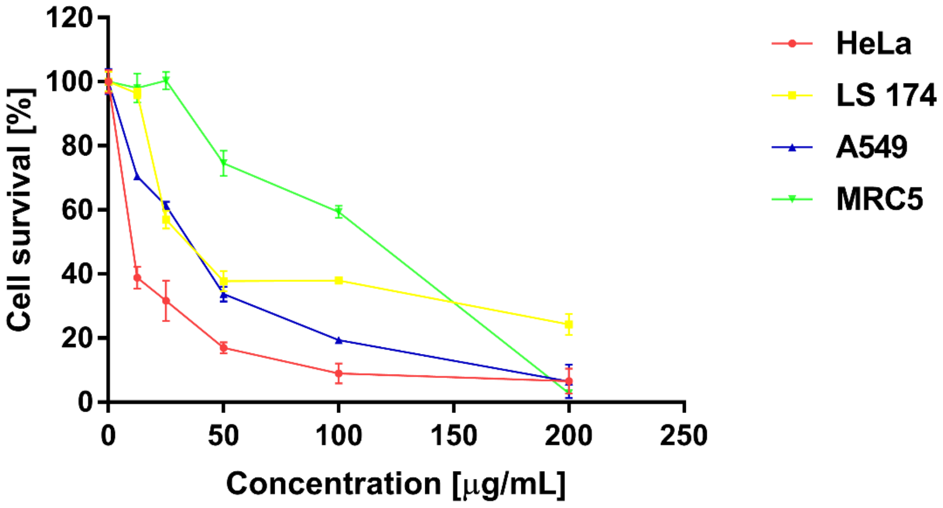

Figure S1. Representative graph of HeLa, LS174, A549 and MRC-5 cells survival after 72 hours of cell growth in the presence of increasing concentrations of investigated avarol

Supplementary material S2

Protocol for studying the effect of avarol on the growth of solid Ehrlich carcinoma (SEC) in mice

Biological object: female mice F1 (CBA×C57BL/6j) at the age of 2-2.5 months, with a body weight of 19-23 g
Method of neoplasia transplantation: s.c. injection of $2.5 \cdot 10^6$ SEC cells in 0.3 mL of medium 199 in the area of the lateral surface of the right thigh
Animal groups and exposure scheme: control group (n = 17) – after SEC inoculation, does not receive any effects; experimental group (n = 16) – from the 7th to the 20th day after SEC transplantation, avarol is administered daily (50 mg/kg, i.p.) as a 0.5% suspension in water for injection.

Table S1. Individual indicators of the SEC volume at different periods after transplantation in mice of experimental groups

Mouse No.	Tumor volume, mm3					
	7 day	10 day	13 day	15 day	18 day	20 day
Control (untreated) mice						
1	92	310	503	681	996	1458
2	47	190	359	681	1040	1256
3	42	117	190	359	567	942
4	92	206	310	679	1048	1331
5	42	117	245	442	605	806
6	47	127	245	385	569	994
7	58	144	245	442	721	850
8	52	309	332	850	992	1256
9	64	138	287	536	992	1269
10	84	288	412	568	1155	1524
11	75	206	310	605	851	1155
12	64	190	288	502	803	1155
13	101	338	482	928	1031	1520
14	51	106	144	383	529	756
15	64	264	359	803	1099	1393
16	42	84	190	310	643	981
17	92	222	385	679	1100	1593
Avarol-treated mice						
18	84	190	288	469	804	995
19	117	190	288	570	1047	1155
20	58	157	245	442	721	897
21	75	145	190	359	721	890
22	58	89	120	275	687	759
23	84	190	245	536	898	945
24	84	190	226	412	721	898
25	64	84	117	287	643	681
26	92	190	359	679	851	898
27	47	117	226	412	605	756
28	58	144	174	383	529	678
29	84	144	245	503	898	1100

30	47	93	117	272	558	672
31	42	84	106	245	503	605
32	84	174	206	359	605	804
33	75	206	288	503	756	1039

Table S2. Individual indicators of the SEC relative growth at different periods after transplantation in mice of experimental groups

Mouse No.	Relative tumor volume, rel. units *					
	7 day	10 day	13 day	15 day	18 day	20 day
Control (untreated) mice						
1	1.00	3.36	5.45	7.39	10.80	15.81
2	1.00	4.03	7.62	14.45	22.06	26.67
3	1.00	2.75	4.48	8.47	13.38	22.22
4	1.00	2.23	3.36	7.36	11.36	14.44
5	1.00	2.75	5.78	10.42	14.27	19.00
6	1.00	2.70	5.20	8.17	12.09	21.11
7	1.00	2.49	4.24	7.65	12.49	14.73
8	1.00	5.97	6.40	16.41	19.14	24.24
9	1.00	2.15	4.48	8.36	15.47	19.80
10	1.00	3.43	4.92	6.77	13.78	18.18
11	1.00	2.73	4.11	8.03	11.28	15.31
12	1.00	2.96	4.48	7.84	12.52	18.00
13	1.00	3.36	4.79	9.23	10.26	15.13
14	1.00	2.07	2.81	7.46	10.32	14.73
15	1.00	4.11	5.60	12.52	17.14	21.73
16	1.00	1.98	4.48	7.30	15.16	23.14
17	1.00	2.41	4.18	7.36	11.93	17.28
Avarol-treated mice						
18	1.00	2.27	3.43	5.60	9.59	11.88
19	1.00	1.63	2.47	4.88	8.98	9.90
20	1.00	2.72	4.24	7.65	12.49	15.55
21	1.00	1.91	2.52	4.76	9.56	11.81
22	1.00	1.55	2.08	4.76	11.91	13.16
23	1.00	2.27	2.92	6.40	10.72	11.28
24	1.00	2.27	2.70	4.92	8.61	10.72
25	1.00	1.31	1.82	4.48	10.03	10.62
26	1.00	2.06	3.90	7.36	9.23	9.74
27	1.00	2.47	4.80	8.75	12.84	16.04
28	1.00	2.49	3.02	6.63	9.17	11.76
29	1.00	1.72	2.92	6.00	10.72	13.13
30	1.00	4.03	4.37	9.38	14.45	19.05
31	1.00	1.98	2.50	5.78	11.85	14.27
32	1.00	2.08	2.46	4.29	7.22	9.59
33	1.00	2.73	3.81	6.67	10.03	13.78

* - indicators for each animal (Table 1) are normalized to the initial (on the 7th day) tumor volume before the start of pharmacological interventions;

Table S3. SEC growth inhibition rates in avarol-treated mice at different follow-up periods

Mouse No.	Tumor inhibition, %					
	7 day	10 day	13 day	15 day	18 day	20 day
Avarol-treated mice						
18	0.0	25.1	29.2	38.7	30.2	37.2
19	0.0	46.2	49.1	46.5	34.6	47.7
20	0.0	10.2	12.4	16.2	9.1	17.8
21	0.0	37.0	48.0	47.8	30.4	37.6
22	0.0	48.9	57.0	47.8	13.3	30.4
23	0.0	25.1	39.7	29.9	22.0	40.4
24	0.0	25.1	44.3	46.1	37.3	43.3
25	0.0	56.9	62.5	50.9	27.0	43.9
26	0.0	31.9	19.6	19.3	32.8	48.5
27	0.0	18.3	1.0	4.2	6.5	15.2
28	0.0	17.7	37.7	27.3	33.2	37.8
29	0.0	43.3	39.7	34.3	22.0	30.6
30	0.0	0.0	9.9	0.0	0.0	0.0
31	0.0	34.8	48.4	36.7	13.7	24.5
32	0.0	31.3	49.3	53.0	47.4	49.3
33	0.0	9.8	21.3	27.0	27.0	27.1

Table S4. Effect of avarol on SEC growth in mice

Time after inoculation, day	Relative tumor volume, rel. units; (M ± SD)		Difference level *	Tumor inhibition, %; (M ± SD)
	Untreated mice (n = 17)	Avarol-treated mace (n = 16)		
7	1.00	1.00	$p = 1.000000$	0.0
10	3.03 ± 0.99	2.22 ± 0.63	$p = 0.003498$	28.8 ± 15.6
13	4.85 ± 1.13	3.12 ± 0.87	$p = 0.000107$	35.6 ± 18.0
15	9.13 ± 2.77	6.15 ± 1.53	$p = 0.000254$	32.9 ± 16.4
18	13.73 ± 3.25	10.46 ± 1.85	$p = 0.000756$	24.1 ± 12.7
20	18.91 ± 3.76	12.64 ± 2.62	$p = 0.000034$	33.2 ± 13.7

* - calculated using the Mann-Whitney U test;

Protocol for studying the effect of avarol on the growth of cervical cancer (CC-5) in mice

Biological object: female mice CBA at the age of 2 months, with a body weight of 19-22 g.

Method of neoplasia transplantation: s.c. injection of a homogenate of 100 mg of tumor tissue in 0.5 mL of medium 199 into the region of the lateral surface of the right thigh

Groups of animals and treatment scheme: control group (n = 24) – after CC transplantation, does not receive any effects; the experimental group (n = 24) - from the 7th to the 18th day after CC inoculation, mice are injected daily with avarol (50 mg/kg, i.p.) in the form of a 0.5% suspension in water for injections. On the 14th day, 5 mice are removed from each group for staged morphological studies.

Table S5. Individual indicators of CC volume at different periods after transplantation in mice of experimental groups

Mouse No.	Tumor volume, mm3					
	7 day	9 day	11 day	14 day	16 day	18 day
Control (untreated) mice						
1	308	636	943	1267	1454	2212
2	436	681	890	1267	1523	2035
3	190	536	1047	1212	1885	2111
4	190	567	605	945	1270	2045
5	158	383	536	898	1270	1731
6	308	838	1039	death		
7	353	866	1267	1734	2212	2668
8	226	679	763	1454	1734	2035
9	174	353	679	1204	1454	2212
10	158	594	803	995	1212	death
11	226	503	679	1212	1523	1882
12	189	492	709	803	1097	1454
13	190	605	803	1331	1523	1810
14	158	594	851	1100	1523	1882
15	226	662	943	1454	1734	2386
16	207	567	605	995	1331	2290
17	190	636	756	death		
18	174	359	567	806	1267	1583
19	266	567	1047	1734	1885	death
20	226	756	838	1212	Taken for research	
21	383	785	981	1267	Taken for research	
22	308	636	890	1385	Taken for research	
23	207	756	1039	1394	Taken for research	
24	383	530	898	1212	Taken for research	
Avarol-treated mice						
25	174	469	605	995	1152	1457
26	144	266	412	803	995	1523
27	243	264	567	995	1394	1593
28	383	567	721	851	1152	1810
29	243	383	412	636	851	1267
30	266	469	594	981	1316	1734
31	266	412	709	1140	1394	2035

32	189	334	354	death		
33	383	567	851	1047	1331	1810
34	402	567	605	851	death	
35	189	334	536	898	1047	1523
36	308	492	530	1077	1508	1882
37	308	636	709	1155	1454	2124
38	334	469	530	995	1385	death
39	266	536	721	1097	1385	2124
40	383	503	636	death		
41	353	662	756	1267	1523	2386
42	131	266	570	719	945	1523
43	324	436	503	851	1212	1270
44	226	567	567	995	Taken for research	
45	190	383	567	943	Taken for research	
46	144	206	442	803	Taken for research	
47	402	436	721	806	Taken for research	
48	353	412	567	943	Taken for research	

Table S6. Individual indicators of the CC relative growth at different times after transplantation in mice of experimental groups

Mouse No.	Relative tumor volume, rel. units *					
	7 day	9 day	11 day	14 day	16 day	18 day
Control (untreated) mice						
1	1.00	2.07	3.06	4.12	4.72	7.18
2	1.00	1.56	2.04	2.91	3.50	4.67
3	1.00	2.82	5.51	6.38	9.92	11.11
4	1.00	2.98	3.18	4.97	6.68	10.76
5	1.00	2.42	3.39	5.67	8.02	10.93
6	1.00	2.72	3.37	death		
7	1.00	2.45	3.59	4.91	6.26	7.55
8	1.00	3.00	3.37	6.43	7.67	9.00
9	1.00	2.03	3.90	6.91	8.35	12.70
10	1.00	3.75	5.07	6.28	7.65	death
11	1.00	2.22	3.00	5.36	6.73	8.32
12	1.00	2.61	3.76	4.26	5.82	7.71
13	1.00	3.18	4.23	7.00	8.01	9.52
14	1.00	3.75	5.37	6.94	9.62	11.88
15	1.00	2.92	4.17	6.43	7.67	10.55
16	1.00	2.73	2.92	4.80	6.42	11.05
17	1.00	3.35	3.98	death		
18	1.00	2.06	3.26	4.63	7.27	9.09
19	1.00	2.14	3.94	6.53	7.10	death
20	1.00	3.34	3.70	5.36	Taken for research	
21	1.00	2.05	2.56	3.31	Taken for research	
22	1.00	2.07	2.89	4.50	Taken for research	
23	1.00	3.65	5.01	6.72	Taken for research	
24	1.00	1.38	2.34	3.17	Taken for research	
Avarol-treated mice						
25	1.00	2.69	3.47	5.71	6.61	8.37
26	1.00	1.84	2.86	5.58	6.91	10.58
27	1.00	1.08	2.33	4.09	5.73	6.55
28	1.00	1.48	1.88	2.22	3.01	4.73
29	1.00	1.57	1.69	2.61	3.50	5.21
30	1.00	1.77	2.24	3.70	4.96	6.53
31	1.00	1.55	2.67	4.30	5.25	7.67
32	1.00	1.77	1.88	death		
33	1.00	1.48	2.22	2.73	3.47	4.73
34	1.00	1.41	1.51	2.12	death	
35	1.00	1.77	2.84	4.76	5.56	8.08
36	1.00	1.60	1.72	3.50	4.90	6.11
37	1.00	2.07	2.30	3.75	4.72	6.90
38	1.00	1.41	1.59	2.98	4.15	death
39	1.00	2.02	2.72	4.13	5.22	8.00
40	1.00	1.31	1.66	death		

41	1.00	1.87	2.14	3.59	4.31	6.75
42	1.00	2.03	4.35	5.49	7.22	11.64
43	1.00	1.34	1.55	2.63	3.74	3.92
44	1.00	2.51	2.51	4.40	Taken for research	
45	1.00	2.01	2.98	4.96	Taken for research	
46	1.00	1.43	3.07	5.58	Taken for research	
47	1.00	1.08	1.79	2.00	Taken for research	
48	1.00	1.17	1.61	2.67	Taken for research	

* - indicators for each animal (Table 5) are normalized to the initial (on the 7th day) tumor volume before the start of pharmacological interventions;

Table S7. Indicators of CC growth inhibition in avarol-treated mice at different observation periods

Mouse No.	Tumor inhibition, %					
	7 day	9 day	11 day	14 day	16 day	18 day
Avarol-treated mice						
25	0.0	0.0	4.8	0.0	7.4	11.6
26	0.0	30.1	21.6	0.0	3.3	0.0
27	0.0	58.9	36.1	23.5	19.8	30.9
28	0.0	43.8	48.4	58.4	57.9	50.1
29	0.0	40.3	53.6	51.1	51.0	45.0
30	0.0	33.0	38.7	30.8	30.6	31.0
31	0.0	41.1	28.9	19.6	26.5	19.0
32	0.0	32.9	48.6	death		
33	0.0	43.8	39.2	48.8	51.3	50.1
34	0.0	46.5	58.8	60.4	death	
35	0.0	32.9	22.1	10.9	22.2	14.6
36	0.0	39.4	52.9	34.5	31.4	35.4
37	0.0	21.6	36.9	29.8	33.9	27.1
38	0.0	46.6	56.5	44.2	41.9	death
39	0.0	23.4	25.6	22.7	27.0	15.5
40	0.0	50.2	54.5	death		
41	0.0	29.0	41.4	32.9	39.6	28.7
42	0.0	23.2	0.0	0.0	0.0	0.0
43	0.0	49.0	57.5	50.9	47.6	58.6
44	0.0	4.8	31.3	17.7	Taken for research	
45	0.0	23.6	18.2	7.2	Taken for research	
46	0.0	45.8	16.0	0.0	Taken for research	
47	0.0	58.9	50.9	62.5	Taken for research	
48	0.0	55.7	56.0	50.1	Taken for research	

Table S8. Effect of avarol on CC growth in mice

Time after inoculation, day	Relative tumor volume, rel. units; (M ± SD)		Difference level *	Tumor inhibition, %; (M ± SD)
	Untreated mice	Avarol-treated mace		
7	1.00<br>n = 24	1.00<br>n = 24	$p = 1.000000$	0.0
9	2.64 ± 0.66<br>n = 24	1.68 ± 0.41<br>n = 24	$p = 0.000002$	36.4 ± 15.3
11	3.65 ± 0.91<br>n = 24	2.32 ± 0.71<br>n = 24	$p = 0.000003$	37.4 ± 17.3
14	5.34 ± 1.29<br>n = 22	3.79 ± 1.20<br>n = 22	$p = 0.000559$	29.8 ± 21.3
16	7.14 ± 1.59<br>n = 17	4.95 ± 1.25<br>n = 16	$p = 0.000337$	30.7 ± 17.4
18	9.47 ± 2.12<br>n = 15	7.05 ± 2.11<br>n = 15	$p = 0.005805$	27.8 ± 18.0

* - calculated using the Mann-Whitney U test;

Supplementary material S3

Protocol for studying the effects of avarol on body weight in mice with solid Ehrlich carcinoma (SEC)

Biological object: female mice F1 (CBA×C57BL/6j) at the age of 2-2.5 months, with a body weight of 19-23 g.
Method of neoplasia transplantation: s.c. injection of 2.5 106 SEC cells in 0.3 mL of medium 199 in the area of the lateral surface of the right thigh
Animal groups and exposure scheme: control group (n = 17) – after SEC inoculation, does not receive any effects; experimental group (n = 16) – from the 7th to the 20th day after SEC transplantation, Avarol is administered daily (50 mg/kg, i.p.) as a 0.5% suspension in water for injection.

Table S9. Individual indicators of body weight of mice of experimental groups at different periods after SEC transplantation

Mouse No.	Mouse body weight, g					
	7 day	10 day	13 day	15 day	18 day	20 day
Control (untreated) mice						
1	21	22	23	24	24	25
2	18	19	20	21	22	23
3	21	21	21	22	23	24
4	20	21	21	22	23	24
5	18	19	20	21	22	23
6	19	19	20	20	21	22
7	20	20	20	21	22	23
8	21	21	21	21	23	22
9	21	20	21	22	23	24
10	20	20	21	22	23	24
11	20	19	20	21	22	23
12	21	21	22	22	23	24
13	21	21	21	22	23	24
14	20	20	21	21	22	23
15	22	22	23	22	23	25
16	20	21	21	22	22	23
17	20	20	21	20	21	22
Avarol-treated mice						
18	20	19	20	21	22	23
19	21	22	23	24	25	25
20	21	20	21	22	22	23
21	21	20	20	21	22	23
22	19	18	19	20	22	23
23	20	19	19	20	21	22
24	20	19	20	21	22	23
25	22	21	21	22	23	24
26	20	19	20	21	21	22
27	20	19	19	20	21	22
28	18	18	18	19	20	21

29	20	19	19	20	21	22
30	20	19	19	20	22	23
31	20	19	19	20	21	22
32	19	19	20	20	21	22
33	20	20	21	21	22	23

Table S10. Individual indicators of the relative body weight of mice of the experimental groups at different periods after SEC transplantation

Mouse No.	Mouse relative body weight, % *					
	7 day	10 day	13 day	15 day	18 day	20 day
Control (untreated) mice						
1	100.0	104.8	109.5	114.3	114.3	119.0
2	100.0	105.6	111.1	116.7	122.2	127.8
3	100.0	100.0	100.0	104.8	109.5	114.3
4	100.0	105.0	105.0	110.0	115.0	120.0
5	100.0	105.6	111.1	116.7	122.2	127.8
6	100.0	100.0	105.3	105.3	110.5	115.8
7	100.0	100.0	100.0	105.0	110.0	115.0
8	100.0	100.0	100.0	100.0	109.5	104.8
9	100.0	95.3	100.0	104.8	109.5	114.3
10	100.0	100.0	105.0	110.0	115.0	120.0
11	100.0	95.0	100.0	105.0	110.0	115.0
12	100.0	100.0	104.8	104.8	109.5	114.3
13	100.0	100.0	100.0	104.8	109.5	114.3
14	100.0	100.0	105.0	105.0	110.0	115.0
15	100.0	100.0	104.5	100.0	104.5	113.6
16	100.0	105.0	105.0	110.0	110.0	115.0
17	100.0	100.0	105.0	100.0	105.0	110.0
Avarol-treated mice						
18	100.0	95.0	100.0	105.0	110.0	115.0
19	100.0	104.8	109.5	114.3	119.0	119.0
20	100.0	95.2	100.0	104.8	104.8	109.5
21	100.0	95.2	95.2	100.0	104.8	109.5
22	100.0	94.7	100.0	105.3	115.8	121.1
23	100.0	95.0	95.0	100.0	105.0	110.0
24	100.0	95.0	100.0	105.0	110.0	115.0
25	100.0	95.5	95.5	100.0	104.5	109.1
26	100.0	95.0	100.0	105.0	105.0	110.0
27	100.0	95.0	95.0	100.0	105.0	110.0
28	100.0	100.0	100.0	105.5	111.1	116.7
29	100.0	95.0	95.0	100.0	105.0	110.0
30	100.0	95.0	95.0	100.0	110.0	115.0
31	100.0	95.0	95.0	100.0	105.0	110.0
32	100.0	100.0	105.3	105.3	110.5	115.8
33	100.0	100.0	105.0	105.0	110.0	115.0

* - indicators for each animal (Table 1) are normalized to the initial (on the 7th day) tumor volume before the start of pharmacological interventions;

Table S11. The effect of Avarol on the dynamics of body weight in mice after SEC inoculation

Time after inoculation, day	Mouse relative body weight, %; (M ± SD)		Difference level *
	Untreated mice (n = 17)	Avarol-treated mace (n = 16)	
7	100.0	100.0	$p = 1.000000$
10	100.9 ± 3.2	96.6 ± 2.9	$p = 0.000471$
13	104.2 ± 3.8	99.1 ± 4.5	$p = 0.003376$
15	106.9 ± 5.3	103.4 ± 3.9	$p = 0.146036$
18	111.6 ± 4.9	108.5 ± 4.4	$p = 0.189292$
20	116.2 ± 5.6	113.2 ± 3.9	$p = 0.166231$

* - calculated using the Mann-Whitney U test;

Protocol for studying the effect of avarol on body weight in mice with cervical cancer (CC-5)

Biological object: female mice CBA at the age of 2 months, with a body weight of 19-22 g.
Method of neoplasia transplantation: s.c. injection of a homogenate of 100 mg of tumor tissue in 0.5 mL of medium 199 into the region of the lateral surface of the right thigh
Groups of animals and treatment scheme: control group (n = 24) – after CC transplantation, does not receive any effects; the experimental group (n = 24) - from the 7th to the 18th day after CC inoculation, mice are injected daily with avarol (50 mg/kg, i.p.) in the form of a 0.5% suspension in water for injections. On the 14th day, 5 mice are removed from each group for staged morphological studies.

Table S12. Individual indicators of body weight of mice of experimental groups at different periods after CC transplantation

Mouse No.	Mouse body weight, g					
	7 day	9 day	11 day	14 day	16 day	18 day
Control (untreated) mice						
1	22	22	22	23	23	24
2	21	21	21	22	23	24
3	24	24	24	26	26	27
4	19	19	19	20	21	24
5	23	22	23	24	24	24
6	27	26	25	death		
7	26	26	27	28	28	28
8	26	26	25	26	28	28
9	23	22	23	24	25	26
10	23	23	22	22	21	death
11	22	22	21	23	23	25
12	18	17	17	18	19	22
13	19	18	19	20	20	22
14	21	22	22	23	23	24
15	23	23	23	24	25	26
16	19	19	19	20	21	23
17	23	23	21	death		
18	16	18	18	19	19	20
19	23	23	24	25	24	death
20	23	24	23	24	Taken for research	
21	21	21	21	22	Taken for research	
22	21	20	20	21	Taken for research	
23	21	22	21	22	Taken for research	
24	24	22	23	25	Taken for research	
Avarol-treated mice						
25	23	22	22	23	24	25
26	21	20	21	22	23	24
27	24	23	24	24	25	26
28	22	21	21	23	23	24
29	23	22	22	23	24	25
30	24	23	23	24	24	25

31	25	25	24	26	27	27
32	25	24	23	death		
33	23	22	23	24	24	26
34	23	22	22	22	death	
35	21	20	20	22	23	24
36	26	25	25	27	27	28
37	24	23	25	25	26	27
38	24	23	24	25	26	death
39	22	21	21	22	24	25
40	24	24	25	death		
41	24	23	24	25	26	27
42	23	23	23	24	25	26
43	22	21	22	23	24	25
44	19	19	20	21	Taken for research	
45	20	20	20	21	Taken for research	
46	24	22	23	25	Taken for research	
47	19	19	20	21	Taken for research	
48	21	20	21	23	Taken for research	

Table S13. Individual indicators of the relative body weight of mice of the experimental groups at different periods after CC transplantation

Mouse No.	Mouse relative body weight, % *					
	7 day	9 day	11 day	14 day	16 day	18 day
Control (untreated) mice						
1	100.0	100.0	100.0	104.5	104.5	109.1
2	100.0	100.0	100.0	104.8	109.5	114.3
3	100.0	100.0	100.0	108.3	108.3	112.5
4	100.0	100.0	100.0	105.3	110.5	126.3
5	100.0	95.7	100.0	104.3	104.3	104.3
6	100.0	96.3	92.6	death		
7	100.0	100.0	103.8	107.7	107.7	107.7
8	100.0	100.0	96.2	100.0	107.7	107.7
9	100.0	95.7	100.0	104.3	108.7	113.0
10	100.0	100.0	95.7	95.7	91.3	death
11	100.0	100.0	95.5	104.5	104.5	113.6
12	100.0	94.4	94.4	100.0	105.6	122.2
13	100.0	94.7	100.0	105.3	105.3	115.8
14	100.0	104.8	104.8	109.5	109.5	114.3
15	100.0	100.0	100.0	104.3	108.7	113.0
16	100.0	100.0	100.0	105.3	110.5	121.1
17	100.0	100.0	91.3	death		
18	100.0	112.5	112.5	118.8	118.8	125.0
19	100.0	100.0	104.3	108.7	104.3	death
20	100.0	104.3	100.0	104.3	Taken for research	
21	100.0	100.0	100.0	104.8	Taken for research	
22	100.0	95.2	95.2	100.0	Taken for research	
23	100.0	104.8	100.0	104.8	Taken for research	
24	100.0	91.7	95.8	104.2	Taken for research	
Avarol-treated mice						
25	100.0	95.7	95.7	100.0	104.4	108.7
26	100.0	95.2	100.0	104.8	109.5	114.3
27	100.0	95.8	100.0	100.0	104.2	108.3
28	100.0	95.5	95.5	104.6	104.6	109.1
29	100.0	95.7	95.7	100.0	104.4	108.7
30	100.0	95.8	95.8	100.0	100.0	104.2
31	100.0	100.0	96.0	104.0	108.0	108.0
32	100.0	96.0	92.0	death		
33	100.0	95.7	100.0	104.4	104.4	113.0
34	100.0	95.7	95.7	95.7	death	
35	100.0	95.2	95.2	104.8	109.5	114.3
36	100.0	96.2	96.2	103.9	103.9	107.7
37	100.0	95.8	104.2	104.2	108.3	112.5
38	100.0	95.8	100.0	104.2	108.3	death
39	100.0	95.5	95.5	100.0	109.1	113.6
40	100.0	100.0	104.2	death		

41	100.0	95.8	100.0	104.2	108.3	112.5
42	100.0	100.0	100.0	104.4	108.7	113.0
43	100.0	95.5	100.0	104.6	109.1	113.6
44	100.0	100.0	105.3	110.5	Taken for research	
45	100.0	100.0	100.0	105.0	Taken for research	
46	100.0	91.7	95.8	104.2	Taken for research	
47	100.0	100.0	105.3	110.5	Taken for research	
48	100.0	95.2	100.0	109.5	Taken for research	

* - indicators for each animal (Table 4) are normalized to the initial (on the 7th day) tumor volume before the start of pharmacological interventions;

Table S14. The effect of avarol on the dynamics of body weight in mice after CC inoculation

Time after inoculation, day	Mouse relative body weight, %; (M ± SD)		Difference level *
	Untreated mice	Avarol-treated mace	
7	100.0<br>n = 24	100.0<br>n = 24	$p = 1.000000$
9	99.6 ± 4.3<br>n = 24	96.6 ± 2.2<br>n = 24	$p = 0.009253$
11	99.3 ± 4.4<br>n = 24	98.7 ± 3.6<br>n = 24	$p = 0.722115$
14	105.0 ± 4.4<br>n = 22	103.8 ± 3.6<br>n = 22	$p = 0.110865$
16	107.1 ± 5.4<br>n = 17	106.5 ± 2.8<br>n = 16	$p = 0.375445$
18	114.7 ± 6.5<br>n = 15	110.8 ± 3.1<br>n = 15	$p = 0.087810$

* - calculated using the Mann-Whitney U test;
